# Iron Supplementation with Ferrous Sulfate or Ferrous Bisglycinate for 12 Weeks Does Not Influence Group B Streptococcus Colonization in Cambodian Women: A Secondary Analysis of a Randomized Controlled Trial

**DOI:** 10.1016/j.tjnut.2025.10.014

**Published:** 2025-10-09

**Authors:** Elisa Cirigliano, Annie Saint, Lulu X Pei, Catherine WY Wong, Siyun Wang, Jordie AJ Fischer, Hou Kroeun, Crystal D Karakochuk

**Affiliations:** 1 Human Nutrition, Faculty of Land and Food Systems, The University of British Columbia, Vancouver, Canada; 2 BC Children’s Hospital and Women’s Health Research Institutes, Vancouver, Canada; 3 Food Science, Faculty of Land and Food Systems, The University of British Columbia, Vancouver, Canada; 4 Helen Keller Intl, Phnom Penh, Cambodia

**Keywords:** bacteria, gene, infection, intake, microbe, iron, dietary supplement, Group B Streptococcus, Cambodia, gene expression

## Abstract

**Background:**

Iron deficiency in women of reproductive age can have severe adverse perinatal consequences. Although iron supplementation can be effective at treating iron deficiency, excess unabsorbed iron in the gut may also promote colonization by enteropathogens such as Group B Streptococcus (GBS), increasing the potential risk of maternal and neonatal infection.

**Objectives:**

We examined whether 12 wk of supplementation with 18 mg elemental iron as ferrous bisglycinate, 60 mg as ferrous sulfate, or a placebo differentially influences GBS colonization in Cambodian women of reproductive age.

**Methods:**

This study is a secondary analysis of a randomized controlled trial conducted in 25 villages in 3 districts of Kampong Thom province, Cambodia, including 144 nonpregnant women (18‒45 y) who received 18 mg of elemental iron as ferrous bisglycinate, 60 mg ferrous sulfate, or placebo for 12 wk. GBS colonization was assessed by real-time quantitative polymerase chain reaction targeting the *cfb* gene (which encodes the Christie-Atkins-Munch-Peterson [CAMP] factor, an indicator of GBS colonization) from stool collected at baseline and 12 wk. Cycle threshold values were compared within and across groups using Kruskal-Wallis and Wilcoxon signed-rank tests.

**Results:**

No changes in *cfb* expression were detected between baseline and endline within any treatment arm, nor were there differences across groups at 12 wk. However, regional differences in *cfb* expression were detected, with participants from the Srayov district exhibiting lower baseline and endline expression, than those from Prey Kuy and Tboung Krapeu.

**Conclusions:**

Oral iron supplementation for 12 wk did not increase *cfb* expression in this population of predominantly iron-replete Cambodian women, compared with placebo. However, the observed geographic variation in *cfb* expression across districts suggests that environmental or behavioral factors may contribute to GBS colonization risk. These findings highlight the need to further investigate region-specific risk factors and provide a foundation for future research into GBS screening in Cambodia.

This trial was registered at clinicaltrials.gov as NCT04017598.

## Introduction

Adequate iron status is important for nonpregnant women of reproductive age who may soon become pregnant, because iron deficiency (ID) in the perinatal period can increase risk of adverse maternal and fetal outcomes, such as postpartum hemorrhage, low birthweight or small for gestational age infants, and stillbirth [1,2]. As such, the World Health Organization (WHO) global guidelines recommend daily oral iron supplementation with 30‒60 mg elemental iron for 12 wk for women and adolescents in regions where anemia prevalence is >40% [3]. Supplemental iron may be beneficial for individuals with ID anemia; however, for those with anemia due to other causes [e.g., hemoglobin (Hb) disorders, inflammation, malarial infection, and/or parasitic infections] [4], additional iron may be harmful.

Evidence has shown that excess unabsorbed iron plays an important role in the virulence and colonization of enteropathogens, such as *Escherichia coli* and *Streptococcus agalactiae*, in the human gut [5,6]. *S*. *agalactiae,* otherwise known as Group B Streptococcus (GBS), is an enteropathogen of concern because it is an opportunistic bacterial pathogen that colonizes the genital and gastrointestinal tracts of many adults and can develop into an invasive disease [7,8]. GBS colonization during pregnancy is a major concern and can lead to complications during or after labor for both the mother and the infant, including amniotic infection, sepsis, meningitis, and death [7,8]. For nonpregnant women, invasive GBS also has several clinical manifestations, including bacteremia, sepsis, pneumonia, soft-tissue infections, and urinary tract infections [[7], [8], [9], [10]]. In many developed countries, such as Canada and the United States, routine GBS screening is performed on pregnant women at 35‒37 weeks of gestation to assess the risk of perinatal GBS transmission [11]. However, in developing countries such as Cambodia, there is currently no policy to screen or treat pregnant women for GBS, and the prevalence of GBS in Cambodian women is largely unknown.

To explore if iron supplementation influences GBS colonization in Cambodian women, we examined the effects of oral iron supplements compared with placebo, specifically whether the form of iron in the supplement (ferrous sulfate compared with ferrous bisglycinate) differentially influences GBS colonization. We conducted real-time qPCR (RT-qPCR) on DNA extracted from stool specimens of Cambodian women to identify the target gene *cfb*, which encodes for the Christie-Atkins-Munch-Peterson (CAMP) factor in GBS [12], as an indicator of GBS colonization. We hypothesized that women who received the iron interventions would have a higher relative increase in *cfb* expression after 12 wk than women who received a placebo.

## Methods

### Study design, study population, and ethical approval

This study was a secondary analysis of a randomized controlled trial of oral iron supplementation in Cambodian women of reproductive age that included 480 women from rural Kampong Thom province [13]. The aims of the original trial were to assess mean ferritin concentrations and other biomarkers after 12 wk of daily oral iron supplementation of 18 mg elemental iron as ferrous bisglycinate, 60 mg ferrous sulfate, or placebo. Key findings of the trial have been published [13]. Ethical approval for the trial was obtained from The University of British Columbia Clinical Research Ethics Board (H18-02610) and the National Ethics Committee for Health Research in Cambodia (273-NECHR). The trial was registered at clinicaltrials.gov (NCT04017598) on 10 July, 2019 and the study protocol has been published [14].

The study population included healthy, nonpregnant women of reproductive age (18‒45 y) living in Kampong Thom, Cambodia, from 25 villages within the 3 districts of Prey Kuy, Srayov, and Tboung Krapeu. Recruitment began in December 2019, baseline visits started in January 2020, and the trial was completed in May 2020. Participants provided written informed consent, and blood and stool specimens were collected at the beginning and end of the 12-wk trial period. Women were excluded if they had consumed antibiotics, dietary supplements, vitamin or mineral supplements, or nonsteroidal anti-inflammatory drugs in the 12 wk prior to the trial. Enrolled participants were randomly assigned at each health center in a 1:1:1 ratio to receive ferrous bisglycinate, ferrous sulfate, or placebo (*n* = 160 for each trial arm) by a computer-generated list of random numbers prepared by the study coordinator (who did not have access to the random allocation sequence).

The trial supplements were produced by Natural Factors (Coquitlam, Canada). The ferrous bisglycinate capsule contained 18 mg elemental iron (experimental treatment), the ferrous sulfate capsule contained 60 mg elemental iron (standard treatment), and the placebo capsule contained microcrystalline cellulose (no elemental iron). All capsules and packaging were identical in size, shape, and color. Natural Factors was responsible for blinding and did not unveil intervention group codes to the statistical analysis team until the analysis for the primary outcome (ferritin concentrations) was completed.

Local research staff in Cambodia conducted regular home visits to monitor adherence to daily supplementation and to record any reported side effects. Trial investigators, research staff, and participants were blinded to the interventions, and the intervention group allocation codes were not unveiled to the research team until the primary outcome analysis was completed.

### Sample collection and processing

Fasting venous blood specimens were collected from participants in the morning on day 1 and again after 12 wk of the intervention. Samples were collected in a 6-mL trace element-free tube, a 6-mL evacuated tube containing EDTA, and a 2-mL tube containing EDTA (Becton Dickinson). A complete blood count was performed using an automated hematology analyzer (Sysmex XN-1000; Sysmex Corporation) to measure Hb (grams per liter) and other hematologic indicators.

Women used an at-home collection kit [15] to obtain a neat stool specimen in their homes, which was transported to the local health center for pick up by the research staff. Neat stool and blood specimens were stored at ‒20°C until shipment on dry ice to The University of British Columbia (Vancouver, Canada), where they were stored at ‒80°C until analysis. Ferritin, α-1-acid glycoprotein, and C-reactive protein were measured using a sandwich-ELISA in Germany [16]. Ferritin was corrected for inflammation using α-1-acid glycoprotein and C-reactive protein concentrations as per globally-endorsed Biomarkers Reflecting the Inflammation and Nutritional Determinants of Anemia guidelines [17].

### RT-qPCR detection of the *cfb* target gene

For the current study, a subset of stool specimens (*n* = 144) was randomly selected from each of the 3 arms using a computer-generated list (*n* = 50 ferrous bisglycinate, *n* = 44 ferrous sulfate, and *n* = 50 placebo). Only *n* = 44 specimens in the ferrous sulfate group were included due to missing baseline or endline matched pairs. DNA was extracted from stool specimens using the QIAamp PowerFecal Pro DNA kit (Qiagen) according to the manufacturer’s instructions. DNA purity was assessed using a nanodrop spectrophotometer (NanoDrop Technologies Inc.). RT-qPCR was performed on DNA extracted from stool specimens to identify the target gene, *cfb*, which encodes for the CAMP factor in GBS [12], as an indicator of GBS colonization. The target gene was amplified from extracted DNA using SsoAdvanced Universal SYBR Green Supermix (Bio-Rad Laboratories Inc.), with primers obtained from Integrated DNA Technologies Inc. (5’-TGGTAGTCGTGTAGAAGCCTTA-3’). To standardize input across reactions, an equal volume of DNA extract was added to each RT-qPCR well, keeping reaction composition constant across specimens. Because DNA concentration varied between stool extracts, the input mass of DNA was not normalized across samples, and results are presented as cycle threshold (Ct) values. All procedures were performed at The University of British Columbia (Vancouver, Canada) following the manufacturer’s instructions.

A 7-point standard curve (10-fold dilutions) was analyzed on each plate to confirm assay linearity and efficiency and to ensure between-plate comparability. Although the standard curve function was applied to Ct values for quality control (QC) purposes, Ct was not converted to absolute copies because *1*) stool extraction yields varied and were not normalized to a fixed stool mass, and *2*) aliquot volumes of DNA, not DNA mass, were used for each reaction. The Ct values indicate the number of cycles needed to replicate enough DNA to be detected [18,19]. The quantity of DNA in the sample is inversely correlated with the Ct value; the higher the quantity of target DNA present in the sample, the lower the Ct value will be [18,19].

### Statistical analyses

The cutoff for *cfb* expression was established in 35 cycles; therefore, specimens with a Ct value <35 were deemed positive for *cfb* expression [19]. Specimens where *cfb* expression was not detected at all were given a Ct value of 40, which is above the threshold for *cfb* detection. This approach was chosen to retain all samples in the analysis, although assigning a fixed Ct value to undetected samples can contribute to a multi-modal distribution. Therefore, median IQR Ct values were reported, and nonparametric statistical tests (Kruskal-Wallis and Wilcoxon signed-ranked tests) were used to compare *cfb* expression across and within groups below. Kruskal-Wallis tests were used to compare *cfb* expression (Ct values) across the 3 treatment arms and between the 3 health center districts at week 0 and week 12 timepoints, with a post hoc Dunn test to determine which groups were significantly different (*P* < 0.01). Paired Wilcoxon signed-rank tests were used to compare differences between week 0 and week 12 within each treatment arm.

## Results

### Participant characteristics

Baseline characteristics of the enrolled cohort by trial arm are reported in Table 1. Women had a mean ± SD age of 34 ± 7 y, a BMI (kg/m^2^) of 23.6 ± 3.7, and a median [IQR] number of children of 2 [1, 3]. Information about the health status of the participants is detailed in Table 2 [16]. At baseline, the prevalence of anemia (Hb <120 g/L) was 17% (*n* = 25/144), ID based on inflammation-adjusted ferritin <15 μg/L was 6% (*n* = 9/144), and ID anemia based on inflammation-adjusted ferritin <15 μg/L and Hb <120 g/L was 3% (*n* = 5/144). Overall, 62% of women (*n* = 90/144) were highly adherent to the supplement regime, defined as consuming 80% or >67 of the 84 prescribed capsules over the 12 wk. Following 12 wk of supplementation, median [IQR] ferritin concentration was 89.4 [61.0, 121.0] μg/L in the ferrous sulfate group, 89.4 [49.2, 120.6] μg/L in the ferrous bisglycinate group, and 85.5 [42.8, 111.8] μg/L in the placebo group, with an ID prevalence of 0%, 0%, and 6% (*n* = 3/50) in each group respectively.

**TABLE 1:** Baseline characteristics of the enrolled Cambodian women by treatment arm.

	18 mg Ferrous bisglycinate (*n* = 50)	60 mg Ferrous sulfate (*n* = 44)	Placebo (*n* = 50)
Age, y	33.2 ± 7.2	35.0 ± 6.6	34.1 ± 7.9
BMI (kg/m^2^)	22.6 ± 3.6	23.6 ± 3.5	24.5 ± 3.8
Parity	2 [2, 3]	2 [1, 3]	2 [1, 3]
Household size	4.8 ± 1.4	4.3 ± 1.2	4.4 ± 1.1
Health center, %			
Prey Kuy, *n* = 46	16/46 (35)	14/46 (30)	16/46 (35)
Srayov, *n* = 51	17/51 (33)	17/51 (33)	17/51 (33)
Tboung Krapeu, *n* = 47	17/47 (36)	13/47 (28)	17/47 (36)
Flush to the septic tank household toilet, %	46 (92)	40 (91)	46 (92)
Water source, %			
Hand pump	30 (60)	29 (66)	29 (58)
Ringwell	12 (24)	8 (18)	11 (22)
Pond/river	7 (14)	3 (7)	5 (10)
Bottled water	1 (2)	4 (9)	4 (8)
Other	0 (0)	0 (0)	1 (2)
Animal(s) living in the home, %	46 (92)	37 (84)	45 (90)
Animal(s) living outside the home, %	28 (56)	24 (55)	27 (54)

Values are *n* (%), mean ± SD, or median [IQR].

Abbreviations: BMI, body mass index; IQR, interquartile range; SD, standard deviation.

**TABLE 2:** Health status of enrolled Cambodian women by treatment arm.

	18 mg Ferrous bisglycinate (*n* = 50)	60 mg Ferrous sulfate (*n* = 44)	Placebo (*n* = 50)
Hemoglobin, g/L	130.7 ± 13.1	128.4 ± 12.7	130.7 ± 11.4
Anemia (Hb <120 g/L), %	9 (18)	8 (18)	8 (16)
Serum ferritin,^1^*μ*g/L	62.6 [40.1, 108.3]	74.7 [32.6, 105.8]	84.7 [52.4, 114.1]
Iron deficiency, ferritin <15 *μ*g/L^1^, %	3 (6)	3 (7)	3 (6)
Iron deficiency anemia, Hb <120 g/L and ferritin <15 *μ*g/L^1^, %	3 (6)	1 (2)	1 (2)
CRP, mg/L	0.37 [0.09, 1.77]	0.50 [0.09, 1.29]	0.50 [0.05, 2.36]
>5 mg/L, %	4 (8.0)	1 (2.3)	6 (12.0)
AGP, g/L	0.60 [0.51, 0.75]	0.51 [0.44, 0.69]	0.58 [0.47, 0.79]
>1 g/L, %	6 (12.0)	2 (4.6)	6 (12.0)
Adherence to supplementation^2^, %	28 (56)	29 (66)	33 (66)
Took antibiotics in last year, %	20 (40)	14 (32)	20 (40)

Values are *n* (%), mean ± SD, or median [IQR].

Abbreviations: AGP, α-1-acid glycoprotein; CRP, C-reactive protein; Hb, hemoglobin; IQR, interquartile range; SD, standard deviation.

1 Serum ferritin values were corrected for inflammation [16].

2 Women were defined as adherent if they consumed ≥80% of the capsules at the week 12 capsule count.

### Changes in *cfb* expression from 0 to 12 wk

**FIGURE 1:**
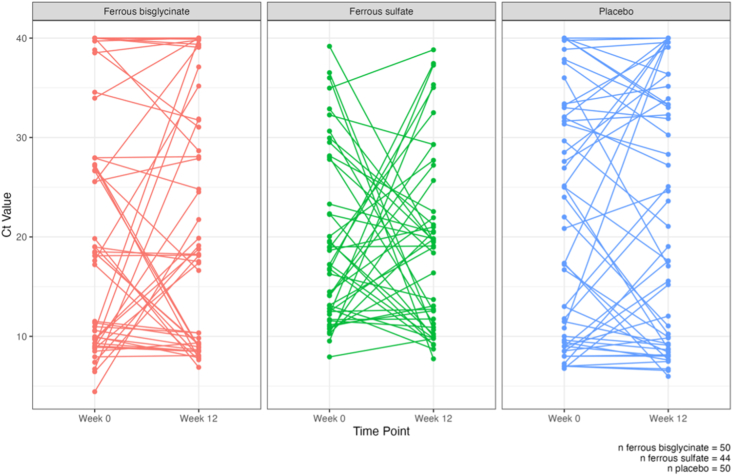
Individual-level change in Ct values (*cfb* gene expression) from week 0 to 12. Ct, cycle threshold.

**FIGURE 2:**
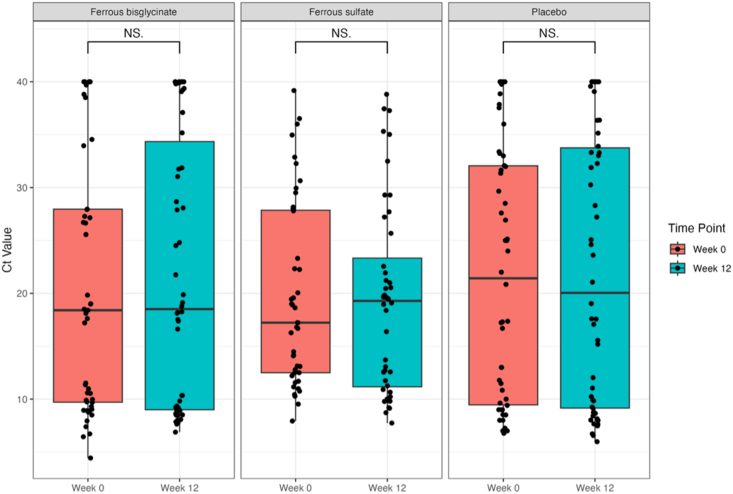
Median [IQR] Ct values (*cfb* gene expression) at weeks 0 and 12 by treatment arm. Ct, cycle threshold; IQR, interquartile range; NS, non-significant.

Figure 1 shows individual-level changes in *cfb* expression (Ct values) from week 0 to week 12, across the 3 trial arms (*n* = 50 ferrous bisglycinate, *n* = 44 ferrous sulfate, and *n* = 50 placebo). Median Ct values for the *cfb* gene were examined for each trial arm between weeks 0 and 12 to determine if the dose and/or form of iron supplementation influences *cfb* expression (Figure 2). The median Ct values for each trial arm at each time point can be found in Table 3. We found no significant differences in *cfb* expression between weeks 0 and 12 in any of the trial arms. Similarly, we also found no significant difference in *cfb* expression between the 3 trial arms at the 12-wk timepoint.

**TABLE 3:** Raw cycle threshold values and proportion of individuals with cycle threshold values <35 at each timepoint by treatment arm.

	18 mg Ferrous bisglycinate (*n* = 50)	60 mg Ferrous sulfate (*n* = 44)	Placebo (*n* = 50)
Ct values at 0 wk	18.4 [9.7, 27.9]	17.22 [12.5, 27.9]	21.4 [9.5, 32.1]
Ct <35 at 0 wk^1^, %	41 (82)	40 (91)	41 (82)
Ct values at 12 wk	18.5 [9.0, 34.3]	19.3 [11.2, 23.3]	20.0 [9.2, 33.8]
Ct <35 at 12 wk^1^, %	37 (74)	39 (89)	38 (76)

Values are *n* (%) or median [IQR].

Abbreviations: Ct, cycle threshold; IQR, interquartile range.

1 A Ct value <35 indicates that *cfb* is present in detectable amounts.

### Regional differences in *cfb* expression

**FIGURE 3:**
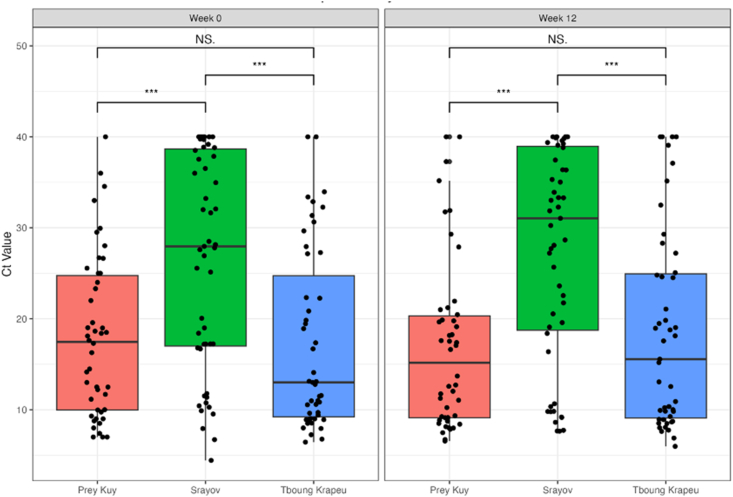
Median [IQR] Ct values (*cfb* gene expression) at weeks 0 and 12 by district. Ct, cycle threshold; IQR, interquartile range; NS, non-significant. ∗∗∗ indicates a statistically signficant difference between the compared groups.

To assess for other risk factors that contribute to GBS in Cambodian women, we also compared *cfb* expression across the 3 districts (Figure 3). Median [IQR] Ct values were 17.5 [10.0, 24.8] at week 0 and 15.2 [9.1, 20.3] at week 12 in Prey Kuy (*n* = 46); 28.0 [17.0, 38.7] at week 0 and 31.0 [18.7, 38.9] at week 12 in Srayov (*n* = 51); and 13.0 [9.2, 24.7] at week 0 and 15.6 [9.1, 24.9] at week 12 in Tboung Krapeu (*n* = 47). Significant differences in Ct values were detected only between Srayov and Prey Kuy, and between Srayov and Tboung Krapeu at both timepoints. At week 0, women from Srayov had significantly higher Ct values (lower *cfb* expression) than women from Prey Kuy (*P* = 0.0014) and Tboung Krapeu (*P* < 0.001). These differences persisted at week 12, with women from Srayov showing lower *cfb* expression than Prey Kuy (*P* < 0.001) and Tboung Krapeu (*P* = 0.001).

## Discussion

Contrary to our hypothesis, 12 wk of daily iron supplementation did not significantly influence *cfb* expression in nonpregnant Cambodian women of reproductive age. Furthermore, no differential effect on *cfb* expression was detected between the 2 forms of iron (ferrous sulfate compared with ferrous bisglycinate), despite differences in the elemental iron dose (ferrous sulfate group receiving 60 mg elemental iron compared with ferrous bisglycinate group receiving 18 mg elemental iron). However, we did observe geographic differences in *cfb* gene expression: women from the Srayov health district had lower *cfb* expression than participants from Prey Kuy or Tboung Krapeu at both 0 wk and 12 wk, suggesting that these differences preceded this trial.

There are several environmental risk factors that have been shown to influence GBS colonization and infection. Previous studies have identified that fish consumption, frequency of sexual intercourse, and panty liner use increased the risk of GBS infection in adults [20,21]. However, the limited data we have available does not allow us to discern the causes of the observed regional differences.

It is important to note the very low prevalence of ID among our study population, especially in consideration of the interpretation of our null findings. Iron homeostasis in the body is tightly regulated by hepcidin, a liver peptide, which controls iron concentrations in the blood through degradation of ferroportin in iron-absorptive enterocytes and iron-recycling macrophages [22]. In iron-replete individuals, hepcidin is upregulated and ferroportin is degraded from the walls of the duodenal enterocytes, which limits iron absorption as a protective mechanism [22,23]. In other words, the absorption of the elemental iron from our study supplements may have been largely inhibited, given our study population was predominantly iron-replete. If this were the case, much of the supplemental iron may have remained unabsorbed and passed into the gut, where it has the potential to fuel the growth of enteropathogens, such as GBS. Thus, although our study population was ideal for testing the hypothesis that unabsorbed iron promotes GBS colonization, we still did not observe significant differences. However, it is possible that a longer duration of supplementation or higher elemental iron doses could yield different results.

Notably, >70% of participants in our study had detectable *cfb* expression (Ct <35) at either baseline or endline, indicating a high prevalence of GBS colonization among healthy, nonpregnant women. This finding has important implications. In many high-income countries, routine screening and intrapartum antibiotic prophylaxis are implemented to prevent vertical transmission of GBS during childbirth. In contrast, no formal screening policies currently exist in Cambodia, and the population-level prevalence of GBS colonization remains poorly characterized. Although our study does not assess active infection or clinical outcomes, the high proportion of *cfb*-positive individuals highlights the need for further research into GBS transmission dynamics and may support future policy discussions around maternal GBS screening in Cambodia.

We acknowledge some limitations in our study. First, the current gold standard for identifying GBS infection set by the Centers for Disease Control and Prevention is rectovaginal sample incubation in a selective medium broth and subculture on a blood agar plate [11]. We acknowledge the limitations in our methodological approach on the use of stool-based RT-qPCR to target the *cfb* gene, because this method cannot distinguish between viable colonization and the transient presence of DNA, and may miss some *cfb*-negative GBS strains. Microbiologic culturing (the clinical gold standard) for GBS would be ideal; however, the feasibility and resources were not available in rural Cambodia to undertake this approach. Several studies have tested the accuracy of RT-qPCR compared with culture techniques to develop a more rapid GBS detection test and have found that RT-qPCR targeting the *cfb*, *sip*, *atr*, and *scpB* genes has proven successful in identifying positive GBS cases in clinical settings [12,[24], [25], [26], [27]]. The *cfb* gene codes for the CAMP factor, which is the primary virulence factor in GBS [12]. Carrillo-Ávila et al. [24] (2018) found that GBS detection using RT-qPCR and the *cfb* target gene alone had a sensitivity of ∼94% and a specificity of ∼95% when compared with cultured samples [24]. However, Zhou et al. [12] (2023) isolated and sequenced GBS strains and found that ∼8% of the strains they isolated were CAMP-negative, concluding that *cfb* alone cannot be the presumptive test for GBS detection [12]. Second, the presence of *cfb* expression in a stool sample is not indicative that the individual had an active GBS infection. GBS colonization can be transient, meaning that colonization status changes over time [28]. Compounding with the fact that we only had 2 collection time points during the 12-wk trial, we were unable to capture short-term fluctuations in *cfb* expression throughout the trial, only the overall change. Despite these limitations, our study has several notable strengths. The use of a randomized controlled trial design with a matched baseline and endline sample for each participant provides a robust framework for assessing longitudinal changes in GBS colonization. The application of RT-qPCR for *cfb* detection enabled sensitive and high-throughput analysis of samples from a low-resource setting where culture-based diagnostics may not be feasible. Moreover, the use of stool specimens allowed for the noninvasive assessment of intestinal colonization, a key reservoir for GBS, and offered a practical alternative to rectovaginal sampling in this rural population. Although *cfb* alone may not capture all GBS strains, it remains a widely used and highly specific molecular marker. In the absence of GBS screening programs in Cambodia, our findings provide valuable foundational data on colonization prevalence and geographic variability among women of reproductive age.

In conclusion, to our knowledge, this is the first published study to examine the effect of iron supplementation on GBS colonization in women in Cambodia. In our cohort of predominantly iron-replete nonpregnant women of reproductive age, we did not detect any differences in GBS colonization across the 2 iron interventions, as compared with placebo. However, we observed significant regional differences in *cfb* expression. Although the estimated prevalence of active GBS infection in women cannot be ascertained from our data, it does raise caution, given the high proportion of individuals with Ct values <35 (indicating the *cfb* gene is present in detectable amounts). Currently, no policy exists in Cambodia to screen pregnant or nonpregnant women for GBS colonization. These findings provide a foundation for future research into GBS screening and disease risk in Cambodia.

## Author contributions

The authors’ responsibilities were as follows– JAJF, HK, CDK: designed the research; JAJF, HK: oversaw specimen collection, transportation and storage; EC, AS, SW: analyzed specimens; EC, LXP, CDK: analyzed data; EC, LXP, CDK: drafted the manuscript; EC, AS, CWYW, SW, JAJF, HK, CDK: contributed to the editing and finalization of the submitted manuscript; CDK: had overall study oversight and primary responsibility for content; and all authors: read and approved the final manuscript.

## Data availability

Data described in the manuscript, code book, and analytic code will be made available upon request pending application and approval by the principal investigator.

## Funding

This project was funded by the Canadian Institutes of Health Research (CIHR) Project Grant (ID400771).

## Conflict of interest

LXP received graduate student scholarships from CIHR and reports a relationship with Balchem Corp that includes: travel reimbursement. JAJF received graduate student scholarships from CIHR. CDK reports a relationship with Balchem Corp that includes: receiving grants. CDK is a Michael Smith Foundation for Health Research Scholar and a CIHR Canada Research Chair in Micronutrients and Human Health. CDK is an editor for The Journal of Nutrition and played no role in the journal’s evaluation of the manuscript. All other authors report no conflicts of interest.

## References

[1] Nair M., Choudhury M.K., Choudhury S.S., Kakoty S.D., Sarma U.C., Webster P. (2016). Association between maternal anaemia and pregnancy outcomes: A cohort study in Assam, India. BMJ Glob. Health.

[2] Shi H., Chen L., Wang Y., Sun M., Guo Y., Ma S. (2022). Severity of anemia during pregnancy and adverse maternal and fetal outcomes. JAMA Netw. Open.

[3] World Health Organization (2011).

[4] Chaparro C.M., Suchdev P.S. (2019). Anemia epidemiology, pathophysiology, and etiology in low- and middle-income countries. Ann. N Y Acad. Sci..

[5] Clancy A., Loar J.W., Speziali C.D., Oberg M., Heinrichs D.E., Rubens C.E. (2006). Evidence for siderophore-dependent iron acquisition in Group B Streptococcus. Mol. Microbiol..

[6] Hantke K., Braun V., Storz G., Hengge-Aronis R. (2000). Bacterial stress responses.

[7] Raabe V.N., Shane A.L. (2019). Group B Streptococcus (Streptococcus agalactiae). Microbiol. Spectr..

[8] Schuchat A. (1999). Group B Streptococcus. Lancet..

[9] Farley M.M., Harvey R.C., Stull T., Smith J.D., Schuchat A., Wenger J.D. (1993). A population-based assessment of invasive disease due to Group B Streptococcus in nonpregnant adults. N. Engl. J. Med..

[10] Skoff T.H., Farley M.M., Petit S., Craig A.S., Schaffner W., Gershman K. (2009). Increasing burden of invasive Group B streptococcal disease in nonpregnant adults, 1990‒2007. Clin. Infect. Dis..

[11] Verani J.R., McGee L., Schrag S.J. Prevention of Perinatal Group B streptococcal disease – revised guidelines from CDC, [Internet] 2010. https://www.cdc.gov/mmwr/preview/mmwrhtml/rr5910a1.html.

[12] Zhou J., Zhang L., Zhang Y., Liu H., Xu K., Zhang B. (2023). Analysis of molecular characteristics of CAMP-negative Streptococcus agalactiae strains. Front. Microbiol..

[13] Fischer J.A., Pei L.X., Elango R., Hou K., Goldfarb D.M., Karakochuk C.D. (2023). Is a lower dose of more bioavailable iron (18-mg ferrous bisglycinate) noninferior to 60-mg Ferrous Sulfate in increasing ferritin concentrations while reducing gut inflammation and enteropathogen detection in Cambodian women? A randomized controlled non-inferiority trial. J. Nutr..

[14] Fischer J.A., Pei L.X., Goldfarb D.M., Albert A., Elango R., Kroeun H. (2020). Is untargeted iron supplementation harmful when iron deficiency is not the major cause of anaemia? Study protocol for a double-blind, randomised controlled trial among non-pregnant Cambodian women. BMJ Open.

[15] Fischer J.A., Karakochuk C.D. (2021). Feasibility of an at-home adult stool specimen collection method in rural Cambodia. Int. J Environ. Res. Public Health..

[16] Erhardt J.G., Estes J.E., Pfeiffer C.M., Biesalski H.K., Craft N.E. (2004). Combined measurement of ferritin, soluble transferrin receptor, retinol binding protein, and C-reactive protein by an inexpensive, sensitive, and simple sandwich enzyme-linked immunosorbent assay technique. J Nutr.

[17] Namaste S.M., Aaron G.J., Varadhan R., Peerson J.M., Suchdev P.S. (2017). BRINDA Working Group, Methodologic approach for the Biomarkers Reflecting Inflammation and Nutritional Determinants of Anemia (BRINDA) project. Am. J Clin. Nutr..

[18] Pabinger S., Rödiger S., Kriegner A., Vierlinger K., Weinhäusel A. (2014). A survey of tools for the analysis of quantitative PCR (qPCR) data. Biomol. Detect. Quantif..

[19] Harshitha R., Arunraj D.R. (2021). Real-time quantitative PCR: A tool for absolute and relative quantification. Biochem. Mol. Biol. Educ..

[20] Barkham T., Zadoks R.N., Azmai M.N., Baker S., Bich V.T., Chalker V. (2019). One hypervirulent clone, sequence type 283, accounts for a large proportion of invasive Streptococcus agalactiae isolated from humans and diseased tilapia in Southeast Asia. PLoS Negl. Trop. Dis..

[21] Esmaon R., Lim B.K., Gan F., Hamdan M., Tan P.C. (2024). Sexual activity, vaginal symptoms, maternal perineal hygiene behavior, and constipation on ano-vaginal colonization of Group B Streptococcus in near term pregnancy. BMC Pregnancy Childbirth.

[22] Camaschella C., Nai A., Silvestri L. (2020). Iron metabolism and iron disorders revisited in the hepcidin era. Haematologica.

[23] Nemeth E., Ganz T. (2023). Hepcidin and iron in health and disease. Annu. Rev. Med..

[24] Carrillo-Ávila J.A., Gutiérrez-Fernández J., González-Espín A.I., García-Triviño E., Giménez-Lirola L.G. (2018). Comparison of qPCR and culture methods for group B Streptococcus colonization detection in pregnant women: evaluation of a new qPCR assay. BMC Infect. Dis..

[25] Mousavi S.M., Hosseini S.M., Mashouf R.Y., Arabestani M.R. (2016). Identification of Group B Streptococci using 16S rRNA, cfb, scpB, and atr genes in pregnant women by PCR, Acta. Med. Iran..

[26] Dmitriev A., Suvorov A., Shen A.D., Yang Y.H. (2004). Clinical diagnosis of group B streptococci by scpB gene based PCR, Indian J Med. Res..

[27] Elbaradie S.M., Mahmoud M., Farid M. (2009). Maternal and neonatal screening for group B streptococci by SCP B gene based PCR: A preliminary study, Indian J Med. Microbiol.

[28] Hansen S.M., Uldbjerg N., Kilian M., Sørensen U.B. (2004). Dynamics of Streptococcus agalactiae colonization in women during and after pregnancy and in their infants. J Clin. Microbiol..

